# Long-Term Cultures of Human Cornea Limbal Explants Form 3D Structures *Ex Vivo* – Implications for Tissue Engineering and Clinical Applications

**DOI:** 10.1371/journal.pone.0143053

**Published:** 2015-11-18

**Authors:** Dóra Júlia Szabó, Agate Noer, Richárd Nagymihály, Natasha Josifovska, Sofija Andjelic, Zoltán Veréb, Andrea Facskó, Morten C. Moe, Goran Petrovski

**Affiliations:** 1 Stem Cells and Eye Research Laboratory, Department of Ophthalmology, Faculty of Medicine, University of Szeged, Szeged, Hungary; 2 Center of Eye Research, Department of Ophthalmology, Oslo University Hospital and University of Oslo, Oslo, Norway; 3 Eye Hospital, University Medical Centre, Ljubljana, Slovenia; Cedars-Sinai Medical Center; UCLA School of Medicine, UNITED STATES

## Abstract

Long-term cultures of cornea limbal epithelial stem cells (LESCs) were developed and characterized for future tissue engineering and clinical applications. The limbal tissue explants were cultivated and expanded for more than 3 months in medium containing serum as the only growth supplement and without use of scaffolds. Viable 3D cell outgrowth from the explants was observed within 4 weeks of cultivation. The outgrowing cells were examined by immunofluorescent staining for putative markers of stemness (ABCG2, CK15, CK19 and Vimentin), proliferation (p63α, Ki-67), limbal basal epithelial cells (CK8/18) and differentiated cornea epithelial cells (CK3 and CK12). Morphological and immunostaining analyses revealed that long-term culturing can form stratified 3D tissue layers with a clear extracellular matrix deposition and organization (collagen I, IV and V). The LESCs showed robust expression of p63α, ABCG2, and their surface marker fingerprint (CD117/c-kit, CXCR4, CD146/MCAM, CD166/ALCAM) changed over time compared to short-term LESC cultures. Overall, we provide a model for generating stem cell-rich, long-standing 3D cultures from LESCs which can be used for further research purposes and clinical transplantation.

## Introduction

Cornea epithelial regeneration is essential for maintaining its transparency and normal vision. The complex epithelial turnover is mediated by cornea limbal epithelial stem cells (LESCs), which are found at the junction between the cornea and the conjunctiva in special niches of the basal cell layer [1, 2]. The LESCs possess self-renewal capacity, being able to regenerate the whole corneal epithelium within 12–24 hours time [3]. Loss of LESCs and/or function due to disease or injury can result in impaired corneal function, neovascularization, conjunctival ingrowth and ultimately loss of vision. LESC deficiency (LESCD) [4]—partial or total, can be treated by restoring the limbal area using biopsies from the patient’s healthy eye or transplanting LESCs harvested from autologous or cadaver donor tissue, then cultured and expanded *ex vivo* [5, 6].

Several groups including ours have isolated, cultured and characterized successfully LESCs–all of these studies describe novel methods for cultivating these cells on different biological and synthetic scaffolds in a medium containing or void of serum or other growth supplements[6–9]. The intrinsic capability of limbal explants to generate viable 3D structures *ex vivo* is hereby shown without the use of scaffolds. We recently defined the surface marker fingerprint of LESCs cultivated as monolayer *ex vivo* over short periods of time (2 weeks)–it consisted of positivity for CD117/c-kit, C-X-C chemokine receptor type 4 (CXCR4), CD144/Vascular Endothelial (VE)-Cadherin, CD146/melanoma cell adhesion molecule (MCAM) and CD166/activated leukocyte cell adhesion molecule (ALCAM) [8].

The present study examines the characteristics of long-term *ex vivo* expanded human cornea LESCs in medium containing serum as the only growth supplement using morphological and immunohistochemical techniques. The study intends to use neither biological or synthetic scaffolds nor special surface treatment for adherence of the explants, except a recently developed technique for gravitational attachment of tissues using widely available viscoelastic material [10]. The stemness status (expression of ATP-binding cassette sub-family G member 2 (ABCG2), cytokeratin (CK/KRT) 15, CK19, Vimentin (Vim)), proliferation and differentiation potential (expression of tumor/transformation-related protein 63 alpha (p63α) and Ki-67, and differentiated corneal epithelial markers such as CK 3 and CK12) and extracellular matrix (ECM) formation potential (expression of Collagen I, IV and V) of the LESCs are being tested in 3D grown samples. Furthermore, the surface marker phenotype of the long-standing LESCs are determined and compared to that of short-term cultivation. The study has relevance to obtaining viable and transplantable 3D tissue explants which can be manipulated with forceps, peeled off easily and stand alone from the ‘mother’ tissue for later use in tissue engineering and clinical applications.

## Materials and Methods

### Limbal explants harvesting

All tissue collection complied with the Guidelines of the Helsinki Declaration and was approved by the Regional and Institutional Research Ethics Committee at the University of Debrecen, Hungary (DE OEC: 3094–2010). Limbal tissue collection was done from cadavers only and Hungary follows the EU Member States' Directive 2004/23/EC on presumed consent practice for tissue collection [11]. Tissues were collected from cadavers within 24 hours of biological death. Before enucleation, the surface of the eye was disinfected by 5% povidone iodine (Betadine, Egis, Budapest, Hungary). The conjunctiva was separated from the limbus with conjunctival scissors. Limbal explants isolation was performed under sterile conditions; small (2x2x0.25mm) rectangular shape tissues were dissected by lamellation mainly from the superior, nasal and inferior parts of the corneo-scleral rim.

### Cell culturing

Limbal explants were plated into 24-well cell culture plates in Dulbecco's Modified Eagle's Medium (DMEM, Sigma-Aldrich, St. Louis, MO, USA), supplemented with 10% Fetal Calf Serum (FCS, Sigma-Aldrich, St. Louis, MO, USA), 200mM/mL L-glutamine (Sigma-Aldrich, St. Louis, MO, USA), 1% Antibiotic/Antimycotic Solution (PAA, Pasching, Austria) and maintained in a humidified 5% CO_2_ in air incubator at 37°C.Adherence to the cell culture plate was assured by a gravitational force from viscoelastic (ProVisc, Alcon, Fort Worth, TX, USA) [10]. The explants and the outgrowing LESCs were cultivated in 1 mL of medium, which was then changed every other day for over 3 months. The explants formed 3D cell layers which could be manipulated or lifted easily from the cell culture plates by fine forceps before fixation and further analysis. Since the cells showed different distribution of surface markers, we defined two structures generated by the cells, referred to as 3D cell outgrowth “proximal” to the explant and 3D cell outgrowth “distal” to the explant.

### Cell viability assay and sterility test

Cell viability was determined by measuring levels of ATP using CellTiter-Glo® (Promega, WI, USA), which shows the presence of metabolically active cells. The reagent was added to the respective wells, containing long-term cultures and incubated according to the protocol provided by the manufacturer. Luminescence was recorded by a LuminoSKan Ascent reader (Thermo Scientific, USA). For trypan blue exclusion test, cells were collected by trypsinization in culturing media; 50 μL cell suspension was mixed with equal parts of trypan blue solution (Sigma Aldrich, MO, USA), and cells were counted in a Hemocytometer (Burker chamber). Standard accredited microbiology laboratory (University of Debrecen) tests were carried out to assure sterility (i.e. negativity) of the long-term cultures for *Mycoplasma* (Mycoalert PLUS Mycoplasma Detection Kit, Lonza, Cat. No.: LT07-710).

### Immunofluorescent staining

Long-standing cultures of human cornea LESCs were collected and fixed in 4% paraformaldehyde after peeling off the cell culture plates. The fixed 3D structures were dehydrated in ascending alcohol series and embedded in paraffin; 3–4 μm thick tissue sections were prepared using a rotary microtome, then mounted onto histological slides. After heat-induced antigen retrieval and blocking, immunofluorescent labelling was performed. The samples were characterized for markers of stemness (ABCG2, CK 15, CK19, Vim), proliferation (p63α, Ki-67), limbal epithelial cells- (CK8/18) and differentiated corneal epithelial cell markers (CK3 and CK12). In addition, extracellular matrix deposition of Collagen I, IV and V was characterized in the samples (**S1 Table** summarizes the primary antibodies and dilutions used for immunofluorescent staining). Fluorescent images were taken by a ZEISS Axio Observer.Z1 (ZEISS, Oberkochen, Germany) microscope. Nuclear staining was performed using 4',6-diamidino-2-phenylindole (DAPI) staining. The quantification of postive cells was carried out using standard ImageJ software by three independent individuals. The number of positive cells on the full field of view were taken into account with the help of nuclear (DAPI) staining. Multiple pictures were taken of each sample and the results averaged out as mean ± standard deviation (SD).

### Immunophenotyping of cells

The immunophenotype of the long-term cultures of limbal explants containing outgrowing cells was determined by flow cytometry. FITC, R-phycoerythrin (PE) and allophycocyanin (APC) conjugated antibodies were used to measure the expression of CD34, CD44, (all from BD Biosciences, San Jose, CA, USA); CD31, CD47,CD90/Thy-1, CD117/c-kit, CD146/MCAM, CD166/ALCAM, CXCR4 (all from R&D Systems, Minneapolis, MN, USA) (for further details refer to **S2 Table**). Samples were measured by FACS Calibur flow cytometer (BD Biosciences Immunocytometry Systems) and data were analyzed using Flowing Software 2.5 (PerttuTerho, Turku Centre for Biotechnology, University of Turku, Finland). For comparison, surface marker expression of short-term (2 weeks) cultivated LESCs was used–the isolation and cultivation of such LESCs were based on a previous study[8].

### Transmission electron microscopy

The samples to be analyzed were fixed in 2% glutaraldehyde in cacodylate buffer (pH 7.4) overnight at 4°C, post-fixed in 1% osmium tetroxide, and dehydrated through a graded series of ethanol up to 100%. The samples were then immersed in propyleneoxide for 20 min and embedded in Epon (Electron Microscopy Sciences, Hatfield, PA). Ultra-thin sections (60–70 nm thick) were cut on a Leica Ultracut Ultramicrotome UCT (Leica, Wetzlar, Germany), stained with uranyl acetate and lead citrate and examined using a Tecnai 12 transmission electron microscope (Phillips, Amsterdam, the Netherlands).

### Statistical analysis

Each experiment was performed at least three times and each sample was tested in triplicates. Data are expressed as mean ± S.D. or SEM. Statistically significant differences were determined by student-t tests, a p-value of 0.05 or less was regarded significant.

## Results

Limbal explants were cultured under adherent conditions over long periods of time (90±10 days). Cells grew out from the explants as monolayer within 2 weeks and then underwent stratification and growth proximal and distal to the donor explant tissue.

**Fig 1:**
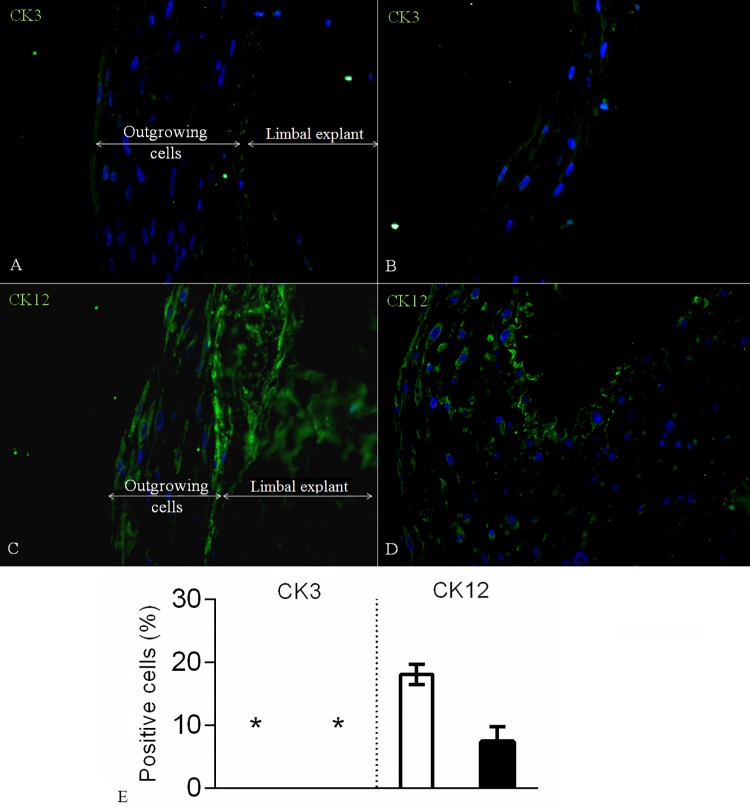
Limbal explants and 3D cell outgrowth stained for differentiation markers. Limbal explants and outgrowing cells proximal (A, C) and distal from it (B, D) being stained for CK3 and CK12, respectively (Magnification: 40x). White bar represents data from limbal explants and proximal outgrowths, while black bar demonstrates data from distal growing 3D cell sheets. (*) means negligible count or zero positive cells expressing CK3. Data are expressed as mean ± SEM.

**Fig 2:**
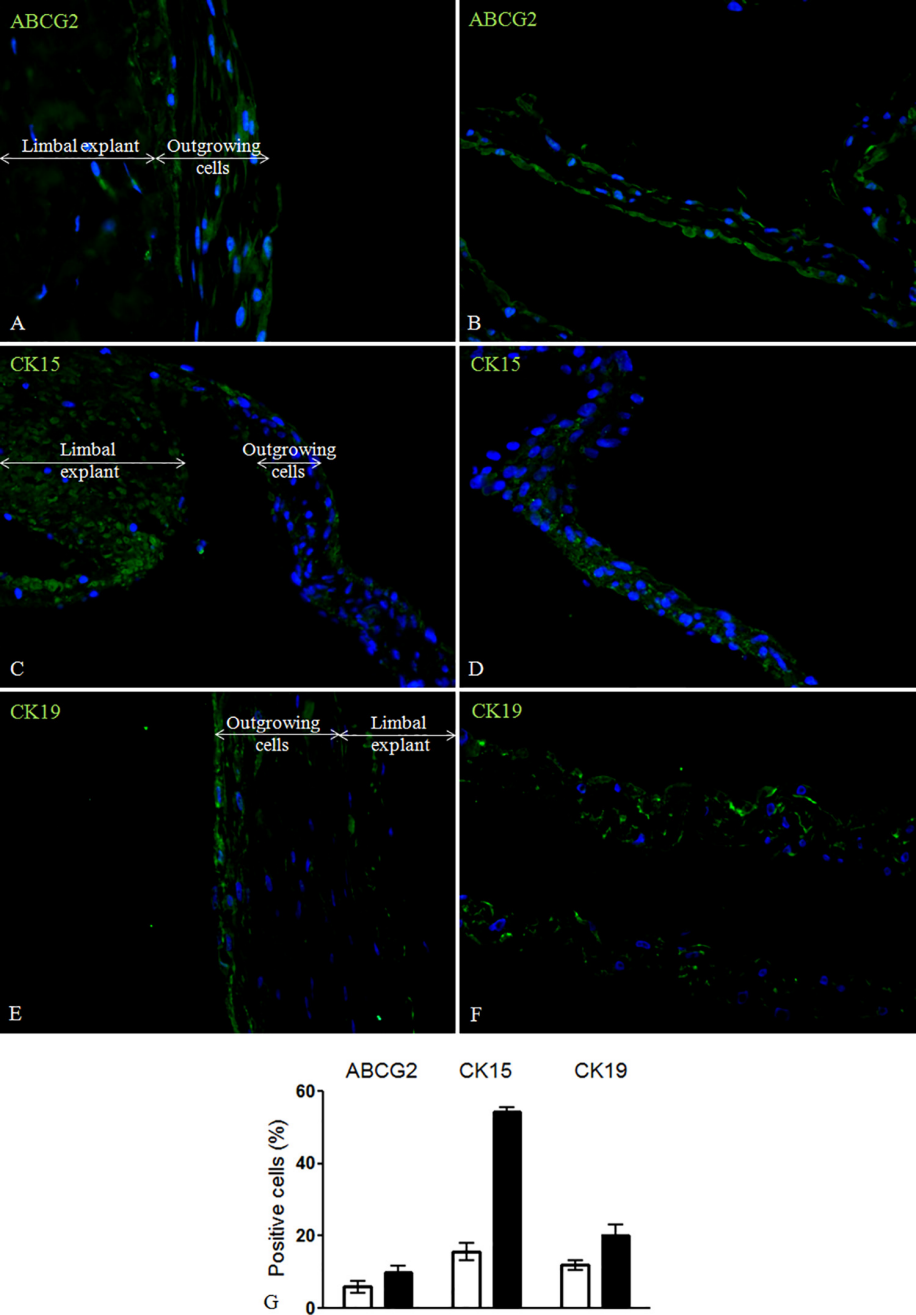
Limbal explants and 3D cell outgrowth stained for stemness markers. Limbal explant and outgrowing cells proximal (A, C, E), and 3D cell outgrowth distal to the explants (B, D, F) being stained for ABCG2, CK15 and CK19, respectively (Magnification: 40x). White bars represent data from limbal explants and proximal outgrowths, while black bars demonstrate data from distal growing 3D cell sheets. Data are expressed as mean ± SEM.

**Fig 3:**
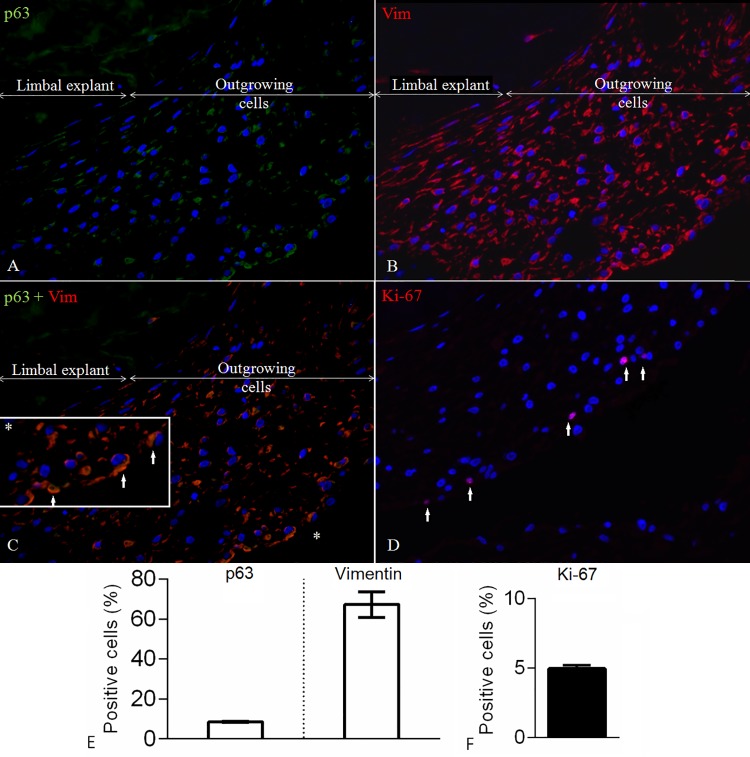
Limbal explants and 3D cell outgrowth stained for additional stemness and proliferation markers. Limbal explant and proximal outgrowing cells stained for p63α and/or Vim (A, B, C), and distal growing cells forming 3D structures being stained for proliferation marker Ki-67 (D) (Magnification: 40x, White bars represent limbal explants and proximal outgrowths, while black bar demonstrate data from distal growing 3D cell sheets. Data are expressed as mean ± SEM.

**Fig 4:**
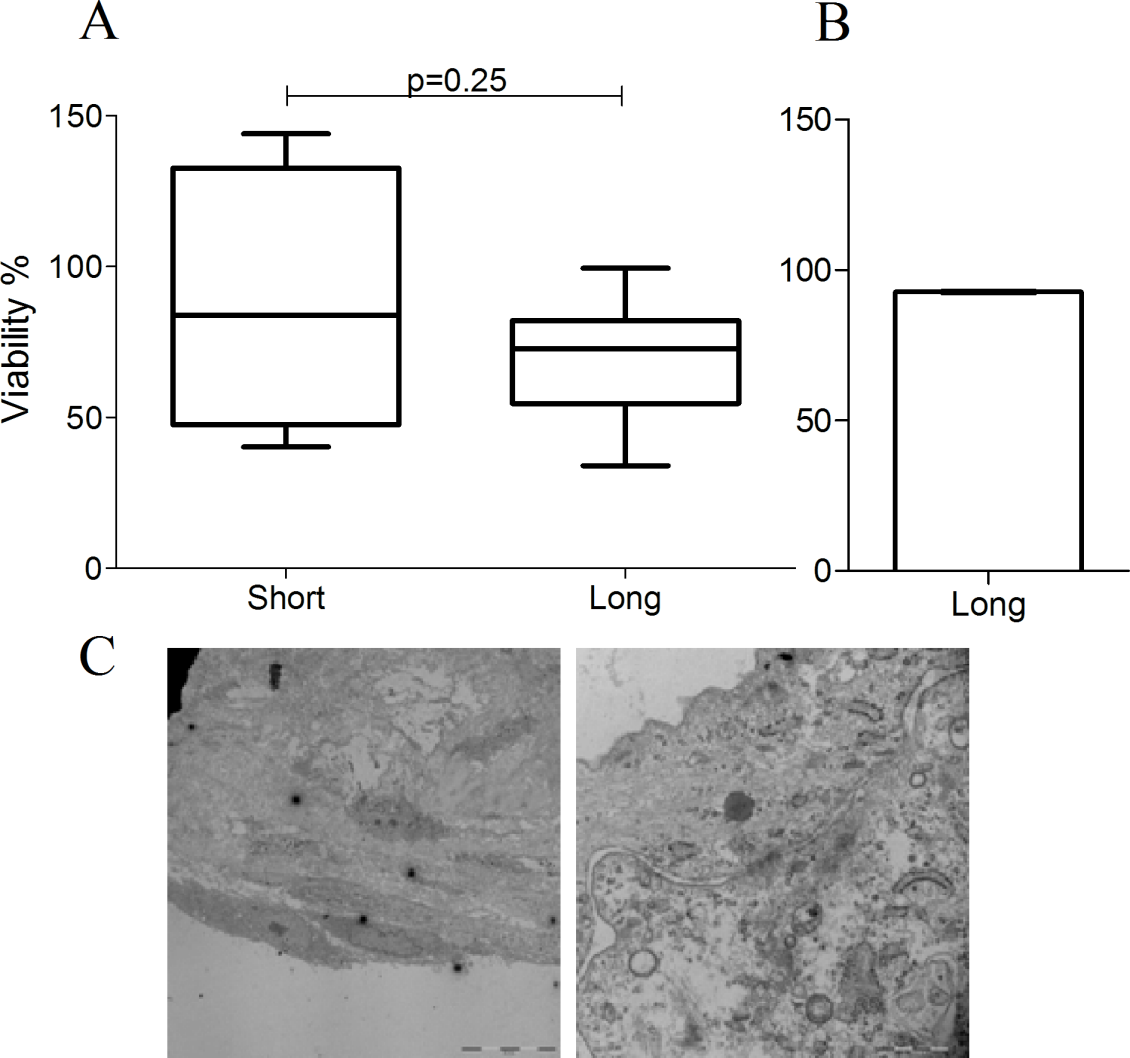
Cellular viability and ultrastructure of the long-term limbal cultures. Tests were performed using CellTiter-Glo® Luminescent assay (A) and Trypan blue exclusion test (B). No significant difference could be observed in the cellular viability between the long- and short-term cultures, based on the ATP level measurements (n = 4). Long-term cultures represented an overall >90% viability (92.76±0.72%, n = 4), when tested by trypan blue exclusion. The ultrastructure of the 3D sheets is shown in (C): left image showing the stratified arrangement of the cells (bar: 20μm); right image showing cell-to-cell-interactions and formation of a desmosome at the center (bar: 1μm).

The differentiation marker CK3 was negative in both samples proximal and distal to the explants. Some CK12 positivity was observed at a close proximity to the explant (18.1±1.6%), as opposed to the distal outgrowing cell sheets (7.4±2.4%) (**Fig 1**).The expression of the stemness marker ABCG2 in the proximal part of the 3D sheets was 6.0^±^1.7%, while in the distal parts its expression was 9.8^±^2.0%. The expression of pluripotency marker CK15 proximal to the limbal explants was 15.6±2.5%, while distal to the explants it was 54.3±1.2%. Similarly, the cytoplasm of the cells growing proximal to the limbal explants was positive for the other more general putative pluripotency marker CK19 in 12.0±1.3%, while in the distal growing 3D sheets it was 20.3±2.7% (**Fig 2**). Separate and co-staining of the limbal explants containing outgrowing cells showed 8.6±0.1% and 67.3±3.7% positivity for the proliferation marker p63α and Vimentin, respectively (**Fig 3**). The proliferation marker Ki-67 was expressed in 5.0±0.1% of the cells. The viability of the long-term cultures was similar to that of short-term cultures and higher than 90% (**Fig 4**). The ultrastructure of the 3D sheets showed stratified nature of the cellular arrangement and cell-to-cell-interactions in the form of desmosomes (**Fig 4**).

**Fig 5:**
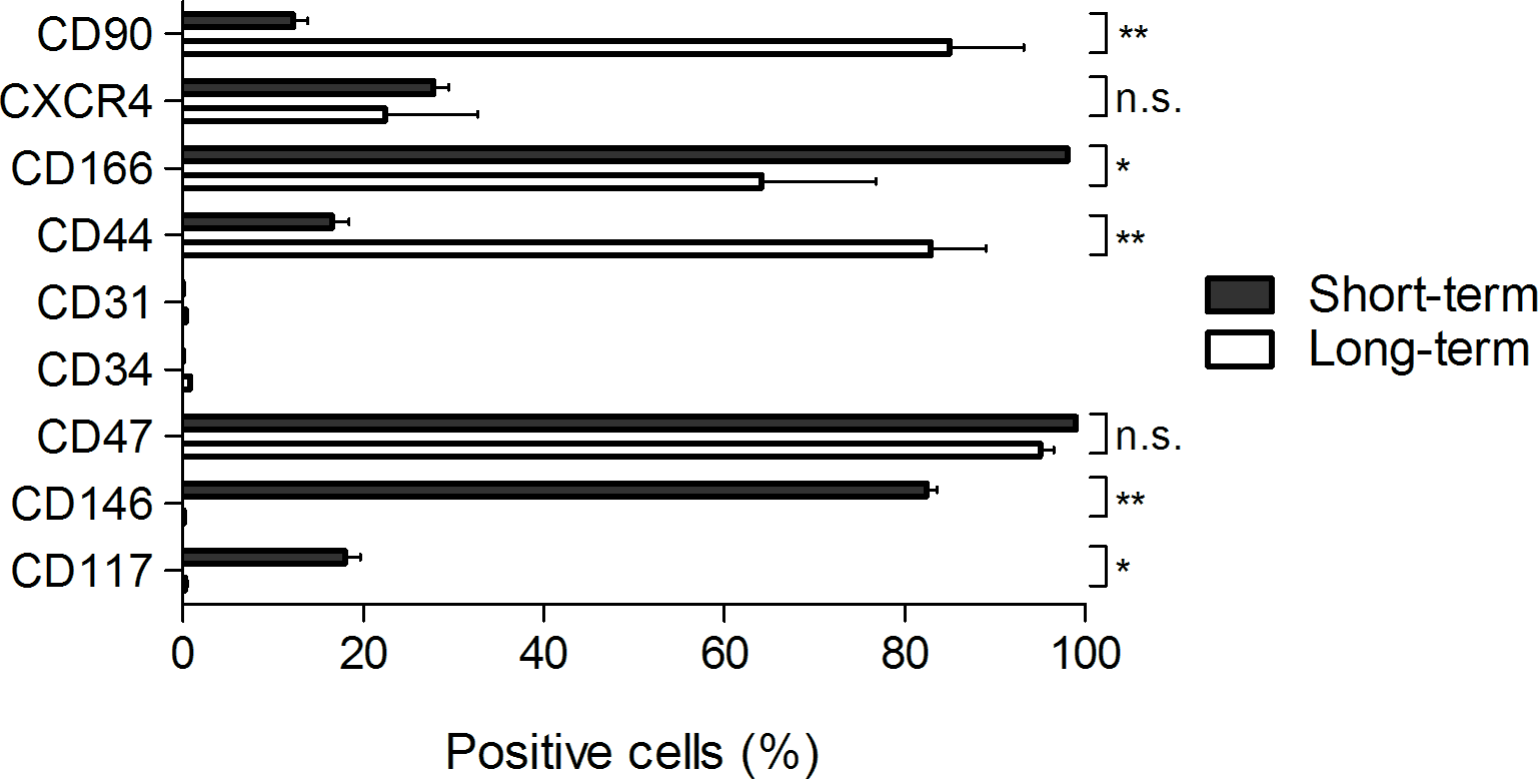
Results of the FACS analyses in long- and short-term limbal cultures. A plot of the percent positive cells in the short- and long-term cultures ± SD is shown. Colors represent different markers measured at different stages of cultivation, the change in expression is demonstrated by the connecting lines.

The surface marker phenotype of the long term outgrowing cells was compared to that of short-term cultures of LESCs (**Fig 5**). The hematopoietic cell surface marker CD34 was not expressed on either the long-term (0.8±0.3%) or the short-term LESCs. The expression of CXCR4 (22.4±10.3%) and CD117/c-kit (0.6±0.1%), characteristic for migrating and early progenitor or pluripotent stem cells, respectively, was abolished in long-term compared to short-term LESC cultures (p = 0.02). A high CD47 expression of long-term LESCs (95.0±2.6%) was similar to that of short-term ones, demonstrating the viability and immunocompetence of both cell types. The endothelial-related marker CD31/Platelet endothelial cell adhesion molecule (PECAM) could not be detected on both cell types, showing no endothelial-related contamination of the cell culture. The change in the expression of extracellular matrix (ECM) attachment proteins, which is important for maintenance of cellular growth in a given milieu was next tested: CD146/MCAM (45.0±63.0%) and CD166/ALCAM (64.16±12.7%) were significantly decreased, while CD44/homing-associated cell adhesion molecule (H-CAM) was significantly increased (73.4±3.1%) (p<0.001) in the long-term LESC cultures compared to the short-term ones. CD144/ VE-Cadherin showed no change over time between the short- and long-term cultures.

**Fig 6:**
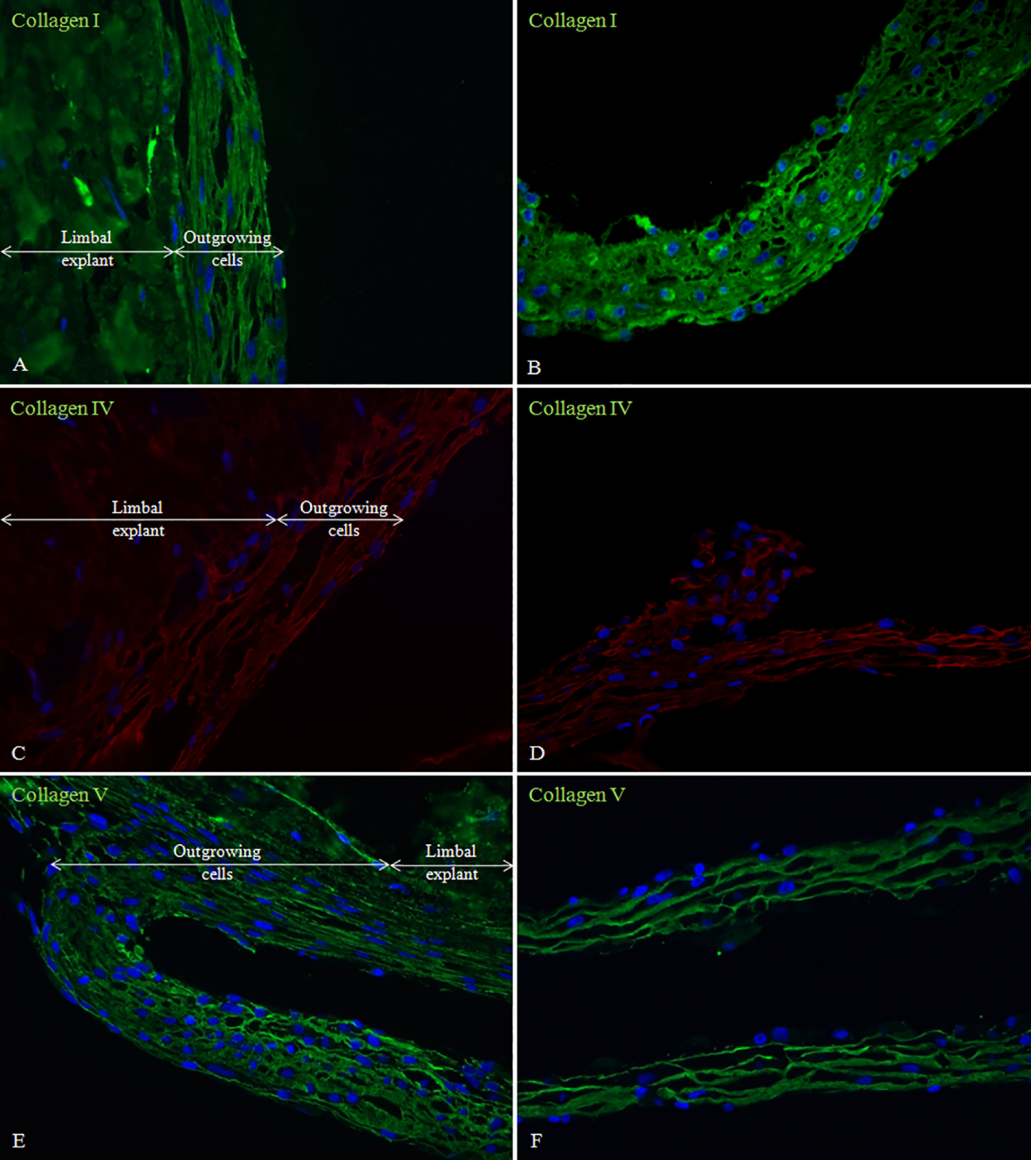
Limbal explants and 3D cell outgrowth stained for extracellular matrix proteins. Limbal explant and proximal outgrowing cells (A, C, E), and 3D cell outgrowth distal to the explants (B, D, F) being stained for collagen I, IV and V, respectively (Magnification: 40x).

The outgrowing cells from the limbal explants formed proximal and distal 3D sheets containing ECM positive for collagen I, IV and V (**Fig 6**)., The Corrected Total Cell Fluorescence (CTCF) of collagen I was 29.1 in the proximal and 35.2 in the distal outgrowing cells, while that of collagen IV and V was 20.9 and 7.8 in the proximal, and 16.6 and 26 in the distal cells from the explants, respectively. These sheets formed a natural scaffold for LESCs to grow into the newly deposited ECM, and could be peeled off easily from the culture plate (**S1 Video**).

## Discussion

Long-term cultures of human cornea limbal explants could be established and characterized morphologically and immunophenotypically *ex vivo*. Viable cell outgrowths of LESCs in the long-term cultures could be kept growing for more than three months without any passaging (longest growth period to date being 9 months). Macroscopically, the LESCs formed thick layers of stratified epithelial tissue, which maintained a well-organized intercellular structure that at termination could be easily lifted by forceps or air-lifting. Most transplantation studies use short-term cultures of LESCs on different biologic or synthetic scaffold, without considering that long-term cultures can make their own scaffold, which can be ideal for transplantations.

The epithelial differentiation marker, CK3, which has been reportedly negative in limbal crypts *in vivo* [1], revealed similar lack of expression in our limbal explants and the outgrowing cells, consistent with the findings by others that, in culture, either the LESCs or the side population expresses CK3, which is gradually lost over time [12]. In contrast, some studies have reported a sporadic [13] or high level of CK3 expression in the superficial versus much less expression in the full thickness limbal tissue-derived cells [14]. Cadaveric limbal samples can indeed have superficial CK3 expression, but not in the basal layers [3]. These results demonstrate that either a CK3 negative population is selected by the depletion of differentiated epithelial cells or differentiated cells revert and lose this marker. By analogy, the terminal differentiation marker CK12 was expressed in one-fifth of the cells proximal to the tissue explants, while the signal was weaker in the 3D cell outgrowth distal to the explants. *In situ* stained limbal cells have been reportedly CK12 negative in the basal layer, although qPCR has revealed low level of expression in the limbal epithelium as opposed to a high expression profile in differentiated corneal epithelium [15]. Our results suggest that the pair of differentiation markers CK3 and CK12 are absent or expressed relatively low in the established long-term LESC cultures, thus the cells are in a relatively undifferentiated state.

ABCG2, an essential component needed for maintenance of a cell’s phenotype, alteration of which can result in depletion of a cell population or commitment to a specific lineage [16], has been shown to be important in upkeeping the stemness of cells in general [16]. A 6.0^±^1.7% expression of this stemness marker was observed proximal to the explants and 9.8^±^2.0% in the 3D cell outgrowth distal to the explants, consistent with data from others [14], while *in vivo* studies have shown ABCG2 positivity at the basal and suprabasal layers [2, 3, 15–18].

CK15, an intermediate filament and pluripotency marker expressed in the basal epithelium in the adult human limbus [19] as well as cultured limbal explant epithelium [20] showed positivity in both the proximal and distal 3D sheets. Similarly, CK19—an intermediate filament protein in epidermal follicle progenitors and a putative stem cell marker [21] being expressed in the basal and peripheral regions of the limbus *in vivo* [1, 13], as well as a gradually increasing marker in explant cultures *in vitro* [12, 14], was positive in 12.0±1.3% of the outgrowing cells proximal to the limbal explants, and much more (20.3±2.7%) in the 3D cell sheets distal to the explants.

The presence of ABCG2, CK15 and CK19 represents a small population of stem/progenitor-like cells in our long-term 3D cultures, which can be a good indicator of their potential for use in clinical transplantation.

An expression of p63α was also present in the cells containing the explants, while the mesenchymal marker Vim was expressed in high quantities under the same conditions. The expression pattern of ABCG2, CK19, Vim and p63α was similar to findings by others regarding the limbal crypt [1, 18], while p63α and Vim appeared to co-localize. The expression of p63α around the explants (8.6±0.1%) can serve as a good indicator for use of these cell sheets in future clinical transplantation. Vim has been found positive in limbal crypts [1, 18] and as highly expressed in cultured limbal explants [14], however, it seems not to be expressed by committed limbal and corneal epithelial cells, thus it serves as a marker of undifferentiated cells [14]. Our samples showed intense staining for Vim around the limbal explants, which serves as a further proof of their stemness. Very likely, these limbal stem cells give rise to a transient amplifying cell population as they mature, but the phenomenon needs further elucidation in the future. The high expression of Vim can probably be explained by the presence of tissue explants or other matrix associations existing in the cultures. The proliferation marker Ki-67 showed low positivity in the distal growing cell sheets, thus, confirming the cells’ low proliferative capacity after 3 months of cultivation. Cultivation of limbal explants for 3 weeks has previously reported a decrease in CK3 expression, while CK19 and Vim were shown to increase over time [12]. This study also found that Ki-67 expression decreased as well, very likely due to reaching confluency of the culturing area. A study with autologous heterotopic transplantation of limbal explants into the central part of the cornea in an animal model resulted in abolishment of p63 positivity, and appearance of CK3 with simultaneous disappearance of Ki-67 expression [22].

Expression studies of progenitor, differentiation, and proliferation markers in limbal epithelial cell cultures from all four limbal regions: superior, nasal, inferior and temporal have found no expression of CK3 in any of the four layers, while strong nuclear p63 expression and cytoplasmic and membranous staining for CK19 and Vim could be noticed in both the basal and suprabasal layers. Furthermore, a moderate membranous staining showing expression of ABCG2 could be detected in all layers of the cultured epithelia. The expression of the proliferation marker Ki67 was not significantly higher in the cultures from the superior limbus compared to the nasal, inferior and temporal cultures [23].

Presence of CD47 on the surface of cells prevents marcophages from engulfing the healthy/viable-marker bearing cells [24–26]. High expression of CD47 in the long-term cultures confirms existence of a viable cell population, which was further confirmed by two different viability assays and presence of healthy ultrastructure of the 3D sheets with cell-to-cell-interactions such as desmosomes.

Although in contrast to the short-term cultured LESCs, where about every fifth or sixth cell expressed CD117/c-kit and every fourth cell expressed CXCR4, in the long-term cultures these markers of proliferation and migration were lost. The lack of room in the culturing wells might contribute to a decline in proliferation and movement as the cell sheets grew thicker.

While the hematopoietic marker CD34 and endothelial marker CD31 were absent in the long-term cultured LESCs, surprisingly, expression of the mesenchymal/ epithelial marker CD90/Thy-1 increased over time to 7-fold in the long-standing cultures versus the short-term ones. Although CD90/Thy-1 has been located on cornea epithelial cells as well [27], its expression on long-term cultures of LESCs might refer to a commitment or a trans-differentiation potential towards epithelial lineage when cultured over longer periods of time. *In situ* presence of CD44/HCAM and CD146/MCAM in the apical layer and CD166/ALCAM in the basal layer have been confirmed by our group. *In vitro*, a 4–4.5-fold increase in the expression of CD44 was observed in the long-term versus short-term cultures.

There was a donor-dependent expression of CD146/MCAM by the long-term LESCs, but the levels of expression did not differ in the short versus long-term cultures. CD166/ALCAM was found to decrease 2.5-fold when cultivation over long periods took place.

Results from the FACS analyses demonstrate that the long-term cultures of LESCs lose the migration and proliferation potential (CD117/c-kit, CXCR4), and either lose (CD166/ALCAM) or upregulate (CD44) adhesion molecules over time and as the milieu changes by the deposition of ECM[28, 29]. However, these cells are viable and even though they show signs of partial differentiation (presence of CK3 and CK12), they express stemness-related markers and are capable of generating transparent tissue with stratified layers of cells resembling *in vivo* structures both macro- and microscopically.

Nuclear staining by DAPI revealed a rather interesting process: the explant cells appeared to migrate out of the donor tissue and toward the cell culture plate (we refer to it here as “empty snail-shell phenomenon”), while at the same time they deposited ECM which was positive for collagen I IV and V. The deposited matrix and the cells growing within its network gave it a stratified appearance similar to the *in vivo* tissue morphology. Heterotopic graft transplantation studies all support presence of focal, milieu- or adhesion matrix-dependent directional expression of pluripotency-to-differentiation markers. Indeed, one member of these ECMs is collagen I, which can organize itself into *de novo* matrix of tightly packed, interconnected type I fibrils with cells growing into multilayers and resembling *in vivo* structures.

Previously, it has been shown that manufactured or compressed collagen I sheets can support limbal cell growth, in a way that both stem- and differentiated cells are present and make up a confluent, multilayered structure, which is ready for transplantation [30]. Similarly, cultured limbal stem cells on collagen Vitrigel could make a stratified epithelium and express p63, while at the same time, matrix associations or presence of Vitrigel support was formed facilitating stemness in the culture dish [31]. Limbal stem cells can grow on laminin-coated compressed collagen containing corneal keratocytes, which provide support for epithelial stratification and formation of transparent tissue [32]. Our data of collagen I expression indicate that LESCs can secrete ECM without any additives or feeder-layer cells to support formation of multi-layered structures. Furthermore, our cultures could synthesize collagen IV and V and demonstrate stratification without any direct induction. We assume that the presence of deposited, insoluble ECM components could serve as a support for the stratification and multilayer-formation.

Overall, we could demonstrate that human limbal tissue explants can give rise to outgrowing cells in a selective medium. The cells can migrate out of the explants and deposit ECM on plastic tissue culture plates, proliferate and form a stratified structure very much like the one *in vivo*. The expanding cells carried proteins related to an undifferentiated state, but also a commitment towards epithelial lineage. Stemness markers suggest a flexible cell phenotype in the long-term cultures. Manipulating the cell sheets was similar to handling primary human tissues, presuming a possibility for use in clinical practice and tissue engineering.

## Supporting Information

S1 Table Details of the antibodies used for immunohistochemistry. (DOCX)

S2 Table List of antibodies used for FACS analysis. (DOCX)

S1 Video Peeling of the 3D structure growing from the limbal explants in the cell culture plates. (MP4)

## References

[1] Shanmuganathan VA, Foster T, Kulkarni BB, Hopkinson A, Gray T, Powe DG, et al Morphological characteristics of the limbal epithelial crypt. The British journal of ophthalmology. 2007;91(4):514–9. 10.1136/bjo.2006.102640 17020899 PMC1994762

[2] Dua HS, Shanmuganathan VA, Powell-Richards AO, Tighe PJ, Joseph A. Limbal epithelial crypts: a novel anatomical structure and a putative limbal stem cell niche. The British journal of ophthalmology. 2005;89(5):529–32. 10.1136/bjo.2004.049742 15834076 PMC1772620

[3] Chang CY, Green CR, McGhee CN, Sherwin T. Acute wound healing in the human central corneal epithelium appears to be independent of limbal stem cell influence. Investigative ophthalmology & visual science. 2008;49(12):5279–86. 10.1167/iovs.07-1260 . 18515566

[4] Pellegrini G, Rama P, Di Rocco A, Panaras A, De Luca M. Concise review: hurdles in a successful example of limbal stem cell-based regenerative medicine. Stem cells. 2014;32(1):26–34. 10.1002/stem.1517 . 24038592

[5] Pathak M, Cholidis S, Haug K, Shahdadfar A, Moe MC, Nicolaissen B, et al Clinical transplantation of ex vivo expanded autologous limbal epithelial cells using a culture medium with human serum as single supplement: a retrospective case series. Acta ophthalmologica. 2013;91(8):769–75. 10.1111/j.1755-3768.2012.02521.x . 22937779

[6] Shahdadfar A, Haug K, Pathak M, Drolsum L, Olstad OK, Johnsen EO, et al Ex vivo expanded autologous limbal epithelial cells on amniotic membrane using a culture medium with human serum as single supplement. Experimental eye research. 2012;97(1):1–9. 10.1016/j.exer.2012.01.013 . 22342952

[7] Lopez-Paniagua M, Nieto-Miguel T, de la Mata A, Galindo S, Herreras JM, Corrales RM, et al Consecutive expansion of limbal epithelial stem cells from a single limbal biopsy. Current eye research. 2013;38(5):537–49. 10.3109/02713683.2013.767350 . 23405945

[8] Albert R, Vereb Z, Csomos K, Moe MC, Johnsen EO, Olstad OK, et al Cultivation and characterization of cornea limbal epithelial stem cells on lens capsule in animal material-free medium. PloS one. 2012;7(10):e47187 10.1371/journal.pone.0047187 23056608 PMC3467238

[9] Gore A, Horwitz V, Gutman H, Tveria L, Cohen L, Cohen-Jacob O, et al Cultivation and characterization of limbal epithelial stem cells on contact lenses with a feeder layer: toward the treatment of limbal stem cell deficiency. Cornea. 2014;33(1):65–71. 10.1097/ICO.0000000000000002 . 24162749

[10] Andjelic S, Lumi X, Vereb Z, Josifovska N, Facsko A, Hawlina M, et al A simple method for establishing adherent ex vivo explant cultures from human eye pathologies for use in subsequent calcium imaging and inflammatory studies. Journal of immunology research. 2014;2014:232659 10.1155/2014/232659 25276840 PMC4168039

[11] European Parliament CotEU. [2015.09.21]. Available from: http://eur-lex.europa.eu/legal-content/EN/ALL/?uri=CELEX:32004L0023.

[12] Joseph A, Powell-Richards AO, Shanmuganathan VA, Dua HS. Epithelial cell characteristics of cultured human limbal explants. The British journal of ophthalmology. 2004;88(3):393–8. 14977776 10.1136/bjo.2003.018481 PMC1772026

[13] Merjava S, Brejchova K, Vernon A, Daniels JT, Jirsova K. Cytokeratin 8 is expressed in human corneoconjunctival epithelium, particularly in limbal epithelial cells. Investigative ophthalmology & visual science. 2011;52(2):787–94. 10.1167/iovs.10-5489 . 20926822

[14] Ghoubay-Benallaoua D, Basli E, Goldschmidt P, Pecha F, Chaumeil C, Laroche L, et al Human epithelial cell cultures from superficial limbal explants. Molecular vision. 2011;17:341–54. 21297898 PMC3033435

[15] Chen Z, de Paiva CS, Luo L, Kretzer FL, Pflugfelder SC, Li DQ. Characterization of putative stem cell phenotype in human limbal epithelia. Stem cells. 2004;22(3):355–66. 10.1634/stemcells.22-3-355 15153612 PMC2906385

[16] Bhattacharya S, Das A, Mallya K, Ahmad I. Maintenance of retinal stem cells by Abcg2 is regulated by notch signaling. Journal of cell science. 2007;120(Pt 15):2652–62. 10.1242/jcs.008417 . 17635990

[17] Priya CG, Prasad T, Prajna NV, Muthukkaruppan V. Identification of human corneal epithelial stem cells on the basis of high ABCG2 expression combined with a large N/C ratio. Microscopy research and technique. 2013;76(3):242–8. 10.1002/jemt.22159 . 23280693

[18] Nubile M, Curcio C, Dua HS, Calienno R, Lanzini M, Iezzi M, et al Pathological changes of the anatomical structure and markers of the limbal stem cell niche due to inflammation. Molecular vision. 2013;19:516–25. 23441125 PMC3580971

[19] Figueira EC, Di Girolamo N, Coroneo MT, Wakefield D. The phenotype of limbal epithelial stem cells. Investigative ophthalmology & visual science. 2007;48(1):144–56. 10.1167/iovs.06-0346 . 17197527

[20] Merjava S, Neuwirth A, Tanzerova M, Jirsova K. The spectrum of cytokeratins expressed in the adult human cornea, limbus and perilimbal conjunctiva. Histology and histopathology. 2011;26(3):323–31. . 21210345 10.14670/HH-26.323

[21] Michel M, Torok N, Godbout MJ, Lussier M, Gaudreau P, Royal A, et al Keratin 19 as a biochemical marker of skin stem cells in vivo and in vitro: keratin 19 expressing cells are differentially localized in function of anatomic sites, and their number varies with donor age and culture stage. Journal of cell science. 1996;109 (Pt 5):1017–28. . 8743949 10.1242/jcs.109.5.1017

[22] Huang M, Wang B, Wan P, Liang X, Wang X, Liu Y, et al Roles of limbal microvascular net and limbal stroma in regulating maintenance of limbal epithelial stem cells. Cell and tissue research. 2014 10.1007/s00441-014-2032-4 . 25398719

[23] Utheim TP, Raeder S, Olstad OK, Utheim OA, de La Paz M, Cheng R, et al Comparison of the histology, gene expression profile, and phenotype of cultured human limbal epithelial cells from different limbal regions. Investigative ophthalmology & visual science. 2009;50(11):5165–72. Epub 2009/07/07. 10.1167/iovs.08-2884 iovs.08-2884 [pii]. . 19578011

[24] Oldenborg PA. CD47: A Cell Surface Glycoprotein Which Regulates Multiple Functions of Hematopoietic Cells in Health and Disease. ISRN hematology. 2013;2013:614619 10.1155/2013/614619 23401787 PMC3564380

[25] McCracken MN, Cha AC, Weissman IL. Molecular Pathways: Activating T Cells After Cancer Cell Phagocytosis from Blockade of CD47 "Don't Eat Me" Signals. Clinical cancer research: an official journal of the American Association for Cancer Research. 2015 10.1158/1078-0432.CCR-14-2520 . 26116271 PMC4621226

[26] Lv Z, Bian Z, Shi L, Niu S, Ha B, Tremblay A, et al Loss of Cell Surface CD47 Clustering Formation and Binding Avidity to SIRPalpha Facilitate Apoptotic Cell Clearance by Macrophages. Journal of immunology. 2015 10.4049/jimmunol.1401719 . 26085683 PMC4490976

[27] Kisselbach L, Merges M, Bossie A, Boyd A. CD90 Expression on human primary cells and elimination of contaminating fibroblasts from cell cultures. Cytotechnology. 2009;59(1):31–44. 10.1007/s10616-009-9190-3 19296231 PMC2677147

[28] Yu FX, Guo J, Zhang Q. Expression and distribution of adhesion molecule CD44 in healing corneal epithelia. Investigative ophthalmology & visual science. 1998;39(5):710–7. . 9538877

[29] Zhu SN, Nolle B, Duncker G. Expression of adhesion molecule CD44 on human corneas. The British journal of ophthalmology. 1997;81(1):80–4. 9135415 10.1136/bjo.81.1.80 PMC1721988

[30] Levis HJ, Menzel-Severing J, Drake RA, Daniels JT. Plastic compressed collagen constructs for ocular cell culture and transplantation: a new and improved technique of confined fluid loss. Current eye research. 2013;38(1):41–52. 10.3109/02713683.2012.725799 . 23016925

[31] McIntosh Ambrose W, Salahuddin A, So S, Ng S, Ponce Marquez S, Takezawa T, et al Collagen Vitrigel membranes for the in vitro reconstruction of separate corneal epithelial, stromal, and endothelial cell layers. Journal of biomedical materials research Part B, Applied biomaterials. 2009;90(2):818–31. 10.1002/jbm.b.31351 . 19283827

[32] Mi S, Chen B, Wright B, Connon CJ. Ex vivo construction of an artificial ocular surface by combination of corneal limbal epithelial cells and a compressed collagen scaffold containing keratocytes. Tissue engineering Part A. 2010;16(6):2091–100. 10.1089/ten.TEA.2009.0748 . 20109018

