# Supplementary material for: Long-Term Cultures of Human Cornea Limbal Explants Form 3D Structures Ex Vivo – Implications for Tissue Engineering and Clinical Applications
Source: PLoS One. 2015 Nov 18;10(11):e0143053. doi: 10.1371/journal.pone.0143053 (PMC4651561; doi:10.1371/journal.pone.0143053)
Supplement: S1 Table — (DOCX) [file pone.0143053.s001.docx]

**S1 Table. Details of the antibodies used for immunohistochemistry.**

| **Antibody** | **Catalog No.** | **Company** | **Clonality** | **Antigen** | **Dilution** |
| --- | --- | --- | --- | --- | --- |
| ABCG2 | Sc-58222 | Santa Cruz | Mouse monoclonal | AA 271-396 of ABCG2 of human origin | 1:50 |
| CK3 | Sc-49179 | Santa Cruz | Goat polyclonal | Peptide mapping at the C-terminus of CK3 of human origin | 1:50 |
| CK8  CK18 | Ab17139 | Abcam | Mouse monoclonal | Cytokeratins from the human breast carcinoma cell line MCF-7 | 1:50 |
| CK12 | Sc-17009 | Santa Cruz | Goat polyclonal | Peptide mapping near the C-terminus of CK12 of human origin | 1:50 |
| CK15 | sc-47697 | Santa Cruz | Mouse monoclonal | Last 17 amino acids of Cytokeratin 15 of human origin | 1:300 |
| CK19 | Sc-33119 | Santa Cruz | Goat polyclonal | Peptide mapping at the N-terminus of CK19 of human origin | 1:50 |
| Collagen I. | Ab34710 | Abcam | Rabbit polyclonal | Human collagen I. AA 1-1464 | 1:200 |
| Collagen IV. | #MS-747-S | Thermo Scientific | Mouse monoclonal | Human glomeruli | 1:50 |
| Collagen V. | Ab7046 | Abcam | Rabbit polyclonal | Full length native protein (purified) corresponding to collagen V. AA 1-1745 | 1:500 |
| Ki-67 | #RM-9106-S | Thermo Scientific | Rabbit monoclonal | Synthetic peptide derived from human Ki-67 protein | 1:200 |
| p63α | Sc-5301 | Santa Cruz | Mouse monoclonal | AA 513-641 of TA*p63a of human origin | 1:50 |
| Vimentin | #RM9120-S | Thermo Scientific | Rabbit monoclonal | Recombinant protein encoding human vimentin | 1:200 |
