# Supplementary material for: Long-Term Cultures of Human Cornea Limbal Explants Form 3D Structures Ex Vivo – Implications for Tissue Engineering and Clinical Applications
Source: PLoS One. 2015 Nov 18;10(11):e0143053. doi: 10.1371/journal.pone.0143053 (PMC4651561; doi:10.1371/journal.pone.0143053)
Supplement: S2 Table — (DOCX) [file pone.0143053.s002.docx]

**S2 Table. List of antibodies used for FACS analysis.**

| **Antibody** | **Clone** | **Catalog No.** | **Company** |
| --- | --- | --- | --- |
| CD34 | 581 | 555821 | BD Biosciences |
| CD44 | BJ18 | 338804 | BD Biosciences |
| CD90/Thy-1 |  | 555595 | BD Biosciences |
| CD31/PECAM | 9G11 | FAB3567P | R&D Systems |
| CD47 | 472603 | FAB4670A | R&D Systems |
| CD117/c-kit | 47233 | FAB332P | R&D Systems |
| CD146/MCAM | 128018 | FAB932A | R&D Systems |
| CD166/ALCAM | 105902 | FAB6561P | R&D Systems |
| CXCR4 | 44717 | FAB173A | R&D Systems |
